# Predatory Publisher Attempts to Compromise *West*JEM’s Integrity

**DOI:** 10.5811/westjem.2018.7.39918

**Published:** 2018-08-08

**Authors:** Mark I. Langdorf

**Affiliations:** University of California, Irvine, School of Medicine, Department of Emergency Medicine, Irvine, California

The advance of Open Access publishing has given rise to a parallel and nefarious process called predatory publishing. Predatory publishing is defined as publishing that “upholds few if any of the best practices, yet demands payment for publishing, even from those most unable to pay.”^1^

As we discussed in our 2016 article (https://escholarship.org/uc/item/64f3v9fj), there are at least 25 to 30 journals related to emergency medicine that engage in predatory practices.^1^

Recently, we became aware that *Arvin Publishers* republished an article from *West*JEM without the author’s approval or acknowledgment of previous publication in *West*JEM (https://escholarship.org/uc/item/47m705bs).

The cornerstone of Open Access publishing is that the copyright remains with the authors and is not assigned to the journal. Therefore, any other journal needs the author’s permission to use all or part of the material.

In this case, *Arvin Publishers* claimed the paper as their own and assigned it to their electronic website as if it had not been previously published. They did not ask for or receive authorization to do this from the paper’s authors.

*West*JEM views this as a serious breach of publication ethics, and sent a letter demanding retraction to *Arvin Publishers.*

Although we received no written response, the plagiarized article was taken down from their website two days after the demand to retract the article was sent. Of note, all five of the articles included in this predatory publisher’s first edition of their emergency medicine journal were stolen from other journals as well.

Predatory publishers use this tactic to appear legitimate with previously published but stolen material masquerading as their own. This can dupe authors into submitting papers for consideration and early publication, only to receive a bill for several thousand dollars after acceptance. These predatory publishers do not provide legitimate editorial services or peer review, and are not indexed in PubMed or any internationally known bibliographic databases, such that other researchers and readers can find the material to learn from and cite.

*West*JEM notified the authors of the other papers so that they could demand retraction as well.

Shortly after we discovered that the plagiarized paper from *West*JEM was removed, we then discovered that the entire current edition of stolen papers has now been taken offline.

The message of this experience is that authors should beware of predatory publishers in general and assure that journals to which they are submitting their work charge reasonable and transparent article processing fees after acceptance. Furthermore, researchers should assure that papers published in a journal are indexed widely so their work receives the attention it deserves.

## References

[1] Hansoti B, Langdorf MI, Murphy LS (2016). Discriminating between legitimate and predatory open access journals: report from the International Federation for Emergency Medicine Research Committee. West J Emerg Med.

